# Continuing Professional Development – Answers

**DOI:** 10.1002/jmrs.828

**Published:** 2024-12-12

Maximise your Continuing Professional Development (CPD) by reading the selected articles and answer the five questions. Please remember to self‐claim your CPD and retain your supporting evidence.

## Answers to Questions Published in Previous Issues

### Volume 70, Issue 4 December 2023

Steward A, Semsem S, Currie K, et al. The cost of perfection: An investigation into the unnecessary rejection of clinically acceptable lateral wrist imaging. *J Med Radiat Sci*. 2023 Dec; 70(4): 380–387. https://doi.org/10.1002/jmrs.702

CPD Questions https:doi/org/10.1002/jmrs.719

**Table:** 

Questions	Answers
1	B
2	D
3	C
4	A
5	C

McCoola B, Outhwaite JA, Lathouras M, et al. An evaluation of the use and efficacy of behavioural therapy when treating paediatric patients with radiation therapy. *J Med Radiat Sci*. 2023 Dec; 70(4): 436–443. https://doi.org/10.1002/jmrs.705

CPD Questions https://doi.org/10.1002/jmrs.728

**Table:** 

Questions	Answers
1	D
2	A
3	B
4	D
5	C

### Volume 71, Issue 1 March 2024

Broadley L, Erskine B, Marshall E, et al. Optimising image quality in intravenous cerebral cone beam computed tomography. *J Med Radiat Sci*. 2024 Mar; 71(1): 26–34. https://doi.org/10.1002/jmrs.735

CPD Questions https://doi.org/10.1002/jmrs.757

**Table:** 

Questions	Answers
1	A
2	C
3	A
4	B
5	C

Forbes E, Clover K, Oultram S, et al. Situational anxiety in head and neck cancer: Rates, patterns and clinical management interventions in a regional cancer setting. *J Med Radiat Sci*. 2024 Mar; 71(1): 100–109. https://doi.org/10.1002/jmrs.736

CPD Questions https://doi.org/10.1002/jmrs.758

**Table:** 

Questions	Answers
1	C
2	A
3	B
4	B
5	D

### Volume 71, Special Issue 2, 2024

Shierlaw E, Penfold M, Crain R, et al. Dosimetric comparison of gantry and horizontal fixed‐beam proton therapy treatment plans for base of skull chordoma. *J Med Radiat Sci*. 2024 Apr;71(Suppl 2):19–26. https://doi.org/10.1002/jmrs.742

CPD Questions https://doi.org/10.1002/jmrs.771

**Table:** 

Questions	Answers
1	A
2	C
3	B
4	D
5	B

Skelton K, Gorayski P, Tee H, et al. Travelling overseas for proton beam therapy: A retrospective interview study. *J Med Radiat Sci*. 2024 Apr; 71(Suppl 2): 10–18. https://doi.org/10.1002/jmrs.721

CPD Questions https://doi.org/10.1002/jmrs.770

**Table:** 

Questions	Answers
1	D
2	B
3	B
4	A
5	C

### Volume 71, Issue 2 June 2024

Girard E, Punch A, Jimenez Y. A wellbeing podcast for diagnostic radiography students. *J Med Radiat Sci*. 2024 Jun; 71(2): 203–213. https://doi.org/10.1002/jmrs.785

CPD Questions https://doi.org/10.1002/jmrs.795

**Table:** 

Questions	Answers
1	C
2	D
3	C
4	B
5	A

Cumming J, Thompson K, Woodford K, et al. The impact of a prophylactic skin dressing on surface‐guided patient positioning in chest wall Radiation Therapy. *J Med Radiat Sci*. 2024 Jun; 71(2): 177–185. https://doi.org/10.1002/jmrs.781

CPD Questions https://doi.org/10.1002/jmrs.799

**Table:** 

Questions	Answers
1	A
2	B
3	D
4	A
5	C

### Volume 71, Issue 3 September 2024

Dolic M, Peng Y, Dhingra K, et al. ePortfolios: Enhancing confidence in student radiographers' communication of radiographic anatomy and pathology. A cross‐sectional study. *J Med Radiat Sci*. 2024 Sep; 71(3): 403–411. https://doi.org/10.1002/jmrs.787

CPD Questions https://doi.org/10.1002/jmrs.805

**Table:** 

Questions	Answers
1	B
2	C
3	B
4	C
5	A

Dower K, Halkett GKB, Dhillon H, et al. Eliciting the views of left breast cancer patients' receiving deep inspiration breath hold radiation therapy to inform the design of multimedia education and improve patient‐centred care for prospective patients. *J Med Radiat* Sci. 2024 Sep; 71(3): 384–395. https://doi.org/10.1002/jmrs.790

CPD Questions https://doi.org/10.1002/jmrs.806

**Table:** 

Questions	Answers
1	D
2	B
3	C
4	B
5	A

